# Development of a model to predict Ki-67 expression status in non-Hodgkin’s lymphoma based on PET radiomics

**DOI:** 10.3389/fonc.2025.1567152

**Published:** 2025-06-18

**Authors:** Ke Tian, Pengfei Tong, Keliu Wu, Amina Azhar, Yuan Fang, Nan Xu, Ruihua Wang

**Affiliations:** ^1^ Department of Radiology, Air Force Medical Center, Air Force Medical University, Beijing, China; ^2^ Department of Neurosurgery, Henan Provincial Third People's Hospital, Zhengzhou, China; ^3^ Department of Nuclear Medicine, Henan Medical Key Laboratory of Molecular Imaging, The First Affiliated Hospital of Zhengzhou University, Zhengzhou, China; ^4^ Imaging and Nuclear Medicine, Zhengzhou University, Zhengzhou, China

**Keywords:** PET/CT, Ki-67, non-Hodgkin lymphoma, invasive, metabolic parameter, radiomics

## Abstract

**Introduction:**

This study aims to evaluate the effectiveness of conventional metabolic parameters and radiomic features from ^18^F-deoxyglucose(FDG) PET in predicting Ki-67 expression status in patients with non-Hodgkin’s lymphoma.

**Methods:**

We analyzed clinical, immunohistochemical(IHC), and ^18^F-FDG PET/CT data from 197 patients diagnosed with non-Hodgkin’s lymphoma at our institution between May 2018 and July 2023. Patients were randomly assigned to a training set (60%) and a validation set (40%) to develop PET image-based radiomics, clinical, and combined models. The models’ predictive abilities were evaluated using receiver operating characteristic (ROC) curves and a nomogram was created to estimate high Ki-67 expression probabilities.

**Results:**

Among the patients, 70 exhibited low Ki-67 expression while 127 had high Ki-67 expression (113 males, 84 females, aged 5–85 years). The high Ki-67 group showed a higher proportion of fever(75.9% vs. 24.1%, P < 0.05) and tumor SUV max value/mediastinal SUV max value (T/MB) (P < 0.01). Five radiomic features formed the radiomics score (AUC: training 0.827; validation 0.883). The combined model showed the highest AUC(training 0.921; validation 0.916), indicating strong predictive capability.

**Conclusion:**

The radiomics model derived from ^18^F-FDG PET demonstrates superior predictive performance for Ki-67 expression status compared to T/MB. The combined model further improves prediction accuracy, highlighting its potential clinical applicability.

## Introduction

Lymphomas are a group of malignant tumors originating in the lymph nodes or other lymphoid tissues and are classified as Hodgkin’s disease (HD) and non-Hodgkin’s lymphoma (NHL). Non-Hodgkin’s lymphoma accounts for 90% of all new lymphoma cases in China each year. The incidence of NHL varies according to age, gender, race, and geographic region. It is characterized by a higher incidence in males than in females, a higher incidence in Caucasians than in other races, a significantly higher incidence in urban than in rural areas, and a higher incidence in developed countries than in developing countries (1–3). Its pathogenesis is associated with environmental factors, lifestyle, immune function abnormalities, viral infections, and genetics (4–6). NHL has been classified into clinically significant aggressive and non-aggressive subtypes (7). Non-aggressive lymphomas, also known as indolent lymphomas, have a slow clinical course and may remain stagnant for years after diagnosis, with an excellent prognosis (8). Although indolent lymphoma is still classified as a malignant tumor, traditional radiotherapy and chemotherapy have limited benefit, and can even have counterproductive effects (9). Aggressive lymphoma progresses rapidly and has a short natural survival period. While there is curative potential through radiotherapy, tumors are prone to recurrence and are difficult to treat (10). Therefore, early identification of lymphoma aggressiveness, precise risk stratification of patients, and risk-based treatment planning can lead to comprehensive, individualized treatment (11, 12).

^18^F-deoxyglucose positron emission tomography/computed tomography (^18^F-FDG PET/CT) not only reveals tumor lesion size but also reflects the metabolic activity within the tumor. Previous studies have demonstrated that measuring semi-quantitative parameters with ^18^F-FDG PET/CT is a simple, non-invasive, and useful method for assessing the proliferative potential of tumor cells (13). In NHL, ^18^F-FDG PET/CT is a clinically recognized and widely used imaging tool for staging and therapeutic evaluation (14–16), and it also plays a valuable role in detecting recurrent disease (17). Non-Hodgkin’s lymphomas are a highly heterogeneous group of malignant tumors of the lymphatic system, and studies on the proliferation and apoptosis of tumor cells have helped to clarify the mechanisms underlying their development. It has been proposed that the varying degrees of FDG uptake in different tumors are related to the differing proliferative activities of the lesions. In lymphomas with low proliferative activity, such as Follicular Lymphoma (FL), chronic lymphocytic leukemia (CLL)/small lymphocytic lymphoma (SLL), and mucosa-associated lymphoid tissue (MALT), where nuclear division is rare, cellular metabolic activity is low, and ^18^F-FDG uptake is minimal. In contrast, lymphomas with high proliferative activity, such as diffuse large B-cell lymphoma (DLBCL), T-cell lymphomas, and lymphoblastic lymphoma, exhibit markedly divided nuclei and high levels of cellular metabolism (18). Ki-67 is a cytosolic antigen expressed only in the nuclei of proliferating cells. In the classification and clinical behavior of lymphoma, Ki-67 serves as an early predictor, not only reflecting tumor aggressiveness and mass size but also showing significant variation in positivity rates across low-, intermediate-, and highly-malignant NHLs. The expression of Ki-67 progressively increases with the degree of malignancy in NHLs (19). In the context of precision diagnosis and treatment, it is crucial to accurately assess Ki-67 expression in diseased lymph nodes by immunohistochemistry before treatment. However, the current clinical approach for detecting Ki-67 expression levels heavily relies on histopathological examination of biopsies or surgical specimens, which is a complex, time-consuming process with many potential interfering factors. This method may lead to biased results due to issues such as insufficient or excessive fixation, antigen repair, and variability in antibody sensitivity during the procedure. Therefore, the need for a non-invasive, simple, comprehensive, and stable assay to predict Ki-67 expression status is a major clinical concern. Unlike biopsy, which is typically limited to a single location, imaging can compare all different lymph node sites throughout the body non-invasively and in multiple consecutive sessions. For example, some low-grade malignant FL may transform into highly malignant DLBCL, which requires immediate identification and initiation of immunotherapy. If an imaging modality can accurately predict the Ki-67 index, it would allow for timely detection and eliminate the limitations of sampling errors. Based on the hypothesis that proliferating tumor cells require more glucose than cells in a resting state, tumors with higher Ki-67 expression may also exhibit higher FDG uptake, as quantified by SUV values. ^18^F-FDG PET and the proliferative index Ki-67 may therefore be correlated (20). However, there is considerable controversy regarding studies on the correlation between FDG uptake and clinical aggressiveness in NHL. Schoder et al. reported that FDG uptake is lower in indolent lymphomas than in aggressive lymphomas, and that patients with a standardized uptake value (SUV) >10 for NHL are more likely to have aggressive disease (21). Luo et al. analyzed 52 NHL patients and concluded that the optimal cutoff value of SUVmax for diagnosing aggressive lymphoma was 12.14 (22). In contrast, Newman et al. found no significant differences in SUV between different sites or grades of NHL across all histologic types in their study (23). There is a lack of sufficiently large validation studies and a need for harmonized methods to define reproducible thresholds.

Radiomics features serve multiple functions, including tumor classification, survival prediction, and treatment response assessment, and play a key role in developing imaging biomarkers for personalized therapy. Radiomics, which is largely independent of the radiologist’s level of experience, can objectively extract additional imaging information to reveal tumor biomarkers, guide specific treatments, track treatment response, detect recurrence, and predict prognosis (24–27). The interdisciplinary field of imaging genomics, which integrates imaging with genomic and molecular data, seeks to analyze the association between genomic variables responsible for phenotypic differences and the corresponding imaging features, providing insights that surpass the limitations of traditional cancer assessment methods (28). Radiomics genomics aims to explore the link between genomic variables and corresponding imaging features. In radiomics, a large volume of quantitative data is extracted from medical images, which may more accurately represent tumor status *in vivo* than SUV alone (29). Recently, new PET texture parameters for histologic assessment and prognosis prediction have emerged, based on the spatial distribution of ^18^F-FDG uptake by lesions (30, 31). Relevant studies exploring how imaging features support histological diagnosis and better report tumor heterogeneity have been reported in breast, lung, gastrointestinal, and renal cancers (32–35). Recently, several studies have highlighted the potential of radiomics features and machine learning-based radiomics models in distinguishing lymphoma from other tumor types in differential diagnosis (36, 37). However, the number of studies focusing on PET to differentiate histologic subtypes of lymphoma and tumor marker expression at the molecular level is limited, and none have elaborated on clinical characteristics (38, 39). Hasan et al. investigated the prediction of lymphoma type using low-dose CT imaging histology obtained from FDG PET-CT, but the study did not evaluate PET data (39). Moreover, the current understanding of Ki-67 expression in lymphomas is limited to conventional PET metabolic parameters, and the predictive value of imaging histology for Ki-67 expression remains unknown. This study aims to develop and validate a radiomics-based PET model for predicting Ki-67 expression, offering a potential non-invasive alternative to biopsy for assessing NHL aggressiveness.

## Materials and methods

### Study design and patients

**Figure 1:**
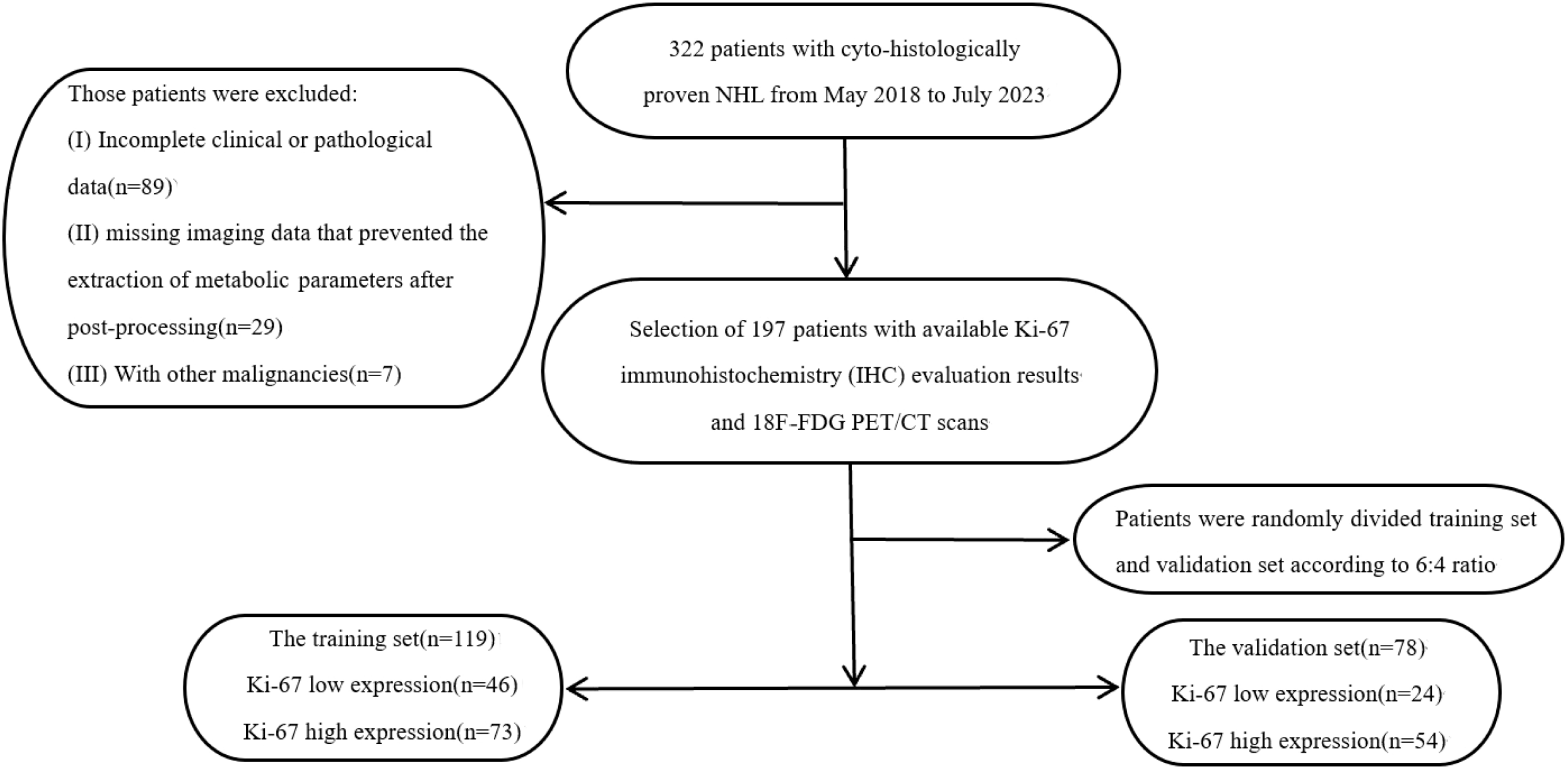
Patient selection and grouping.

Imaging data and clinical information of lymphoma patients who underwent ^18^F-FDG PET/CT imaging at the First Affiliated Hospital of Zhengzhou University from May 2018 to July 2023 were retrospectively collected. The patients were divided into a training group and a validation group in a 6:4 ratio using simple random sampling. Patients were screened based on the following enrollment criteria: (1) all cases were pathologically confirmed as NHL; (2) there was clear histopathological typing; (3) all patients were diagnosed by pathological and immunohistochemical examination, with a report of Ki-67 enzymatic staining for tumor proliferative antigen; (4) ^18^F-FDG PET/CT was performed before pathological sampling, with an interval of no more than 4 weeks; (5) there was no history of other malignant tumors; (6) all patients had primary NHL and had not received prior treatment. Exclusion criteria included: (1) incomplete clinical or pathological data; (2) missing imaging data that prevented the extraction of metabolic parameters after post-processing. A flow diagram of patient selection is shown in **Figure 1**. The study was approved by the institutional review board.

### Data collection and definition

Clinical information was collected for each patient, including gender, age, fever, lymph node involvement, smoking history, and laboratory test results. Superficial lymph node involvement refers to the neck, axilla, and inguinal regions, while deep involvement includes the abdominal cavity, retroperitoneum, mediastinum, and the periphery of the great vessels. The presence or absence of extra-nodal involvement, including bone marrow invasion, was determined through pathological diagnosis. Fever was defined as a temperature >37.3°C before consultation and/or on admission. The normal range for white blood cell count was (4-10) × 10^9^/L; none of the included patients had received treatment, so elevated or decreased white blood cell counts were considered abnormal. Neutropenia was defined as an absolute neutrophil count <1.5 × 10^9/L. Elevated C-reactive protein (>10 mg/L), elevated calcitonin (>0.046 ng/mL), and elevated blood sedimentation rate (>20 mm/h) were also considered abnormal.

The results of Ki-67 enzymatic staining on the immunohistochemistry report were evaluated based on the degree of nuclear staining of tumor cells and the percentage of positive cells. The percentages were classified into five grades, from least to most: 0 (no stained tumor cells), 1 (1%-4% positive cells), 2 (5%-19% positive cells), 3 (20%-49% positive cells), and 4 (≥50% positive cells). A score of 4 was considered high expression, while scores ≤3 were considered low expression.

### PET/CT imaging and data processing

All patients underwent ^18^F-FDG PET/CT using a Siemens Biograph TruePoint (52-ring) PET/CT scanner from Germany. The radiotracert, ^18^F-FDG, was prepared by the Department of Nuclear Medicine at the First Affiliated Hospital of Zhengzhou University, with a radiochemical purity ≥95%. Patients fasted for more than 6 hours and maintained blood glucose levels ≤11.1 mmol/L before ^18^F-FDG PET/CT scanning. ^18^F-FDG was injected intravenously at a dose of 3.70-4.44 MBq/kg (0.10-0.12 mCi/kg), and scanning was performed 45–60 minutes after injection while the patient rested in a quiet, light-protected environment. Whole-body scanning was performed. The bladder was emptied before scanning, and the patient was positioned supine. The acquisition area ranged from the top of the skull to the middle and upper part of the femur. CT scanning was performed first, with the following parameters: tube voltage of 120 kV, tube current of 380 mA, and layer thickness of 3 mm. 3D PET acquisition followed immediately after CT scanning, covering the same area, with a scanning time of 3 minutes per bed for the head and 2.5 minutes per bed for the body, for a total of 4–6 beds. PET data were iteratively reconstructed using the CT data for attenuation correction, and whole-body transverse, coronal, and sagittal CT, PET, and PET/CT fusion images were obtained.

PET/CT images were analyzed, and metabolic parameters, including SUVmax of lymph nodes, metabolic tumor volume (MTV), and total lesion glycolysis (TLG), were measured using a Syngo.via workstation from Siemens, Germany. All PET/CT images were independently analyzed and manually corrected for automatic segmentation results by two nuclear medicine physicians with three and five years of experience, respectively. In cases of disagreement, the final judgment was made by another nuclear medicine physician with more than 10 years of experience in interpreting PET/CT images after review. SUVmax was used to analyze the maximum SUV value across all lesions in the patient’s body. The maximum SUV value of the blood pool in the aortic arch and mediastinal great vessels was also determined, and the T/MB (tumor SUVmax value/mediastinal SUVmax value) ratio was calculated. Two methods were used to outline the regions of interest (ROIs) of the lesions: (i) absolute threshold method (Th2.5): all voxels with standard uptake value (SUV) >2.5 were considered as ROIs; (ii) relative threshold method (Th42%) was used to outline the ROIs of the lesions: all voxels with SUV >42% of SUVmax were considered as ROIs. The dual-threshold approach aimed to validate robustness across methodologies and assess both total tumor burden and high-activity tumor cores. MTV and TLG were calculated using the program provided with the workstation. The total MTV for each patient’s lymph node lesions was defined as the sum of the MTVs of all lymph node lesions (TMTV). The TLG for each lymph node lesion was defined as the product of the SUVmean and MTV of that lesion (TLG = SUVmean × MTV), and the total TLG for a patient’s lymph nodes was defined as the sum of the TLGs of all lymph nodes (TTLG).

### Extraction of PET radiomics parameters

**Figure 2:**
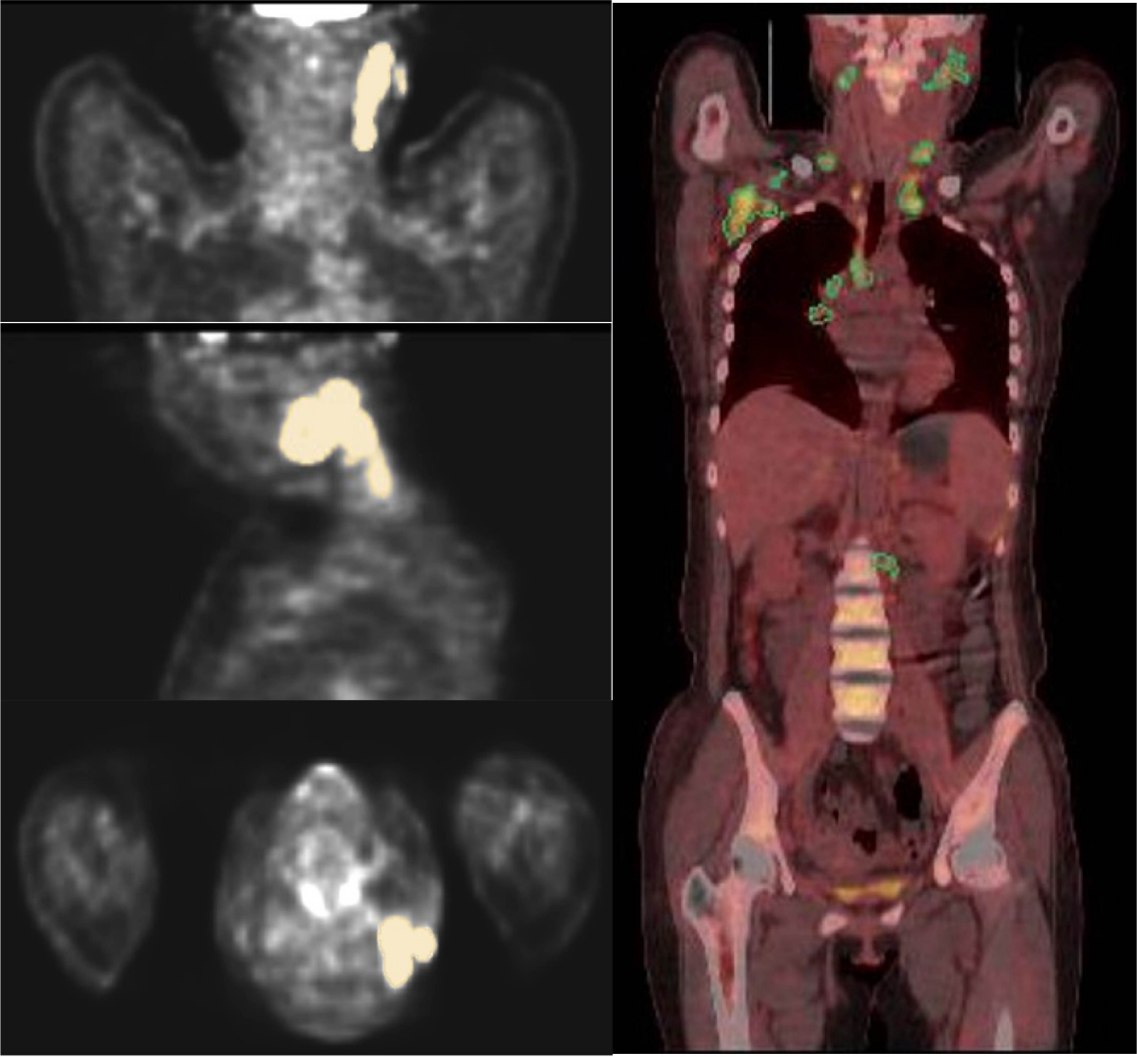
ROIs were outlined layer by layer along the boundary of the lesion, and the figure shows the VOIs outlined in coronal, sagittal, and transverse positions.

The dcm2niix software

(https://www.nitrc.org/projects/dcm2nii/)

was used to convert the DICOM files of PET/CT image data from all collected cases into NIfTI (nii) format files. The volume of interest (VOI) for the lesion regions in the PET images was outlined, and the VOI was adjusted using the CT images as a reference. Only the PET radiomics features were used for analysis. 3D Slicer (http://www.slicer.org) software was used to segment the lesion areas volumetrically on sagittal, transverse, and coronal PET images using the semi-threshold segmentation method. The lesion areas were outlined to coincide with the biopsy site (**Figure 2**). Radiomics features were extracted using the Radiomics module. The original image was processed using a Log (Laplacian of Gaussian) filter and wavelet, and further radiomics features were extracted from both the filtered and original PET images. Additional types of features could be extracted from both the derived and original images, while shape features were extracted exclusively from the original image.

### Screening and modeling of radiomics parameters

(1) Radiomics features with significant differences were calculated using the Mann-Whitney U rank sum test, with the significance threshold set at P < 0.001. (2) The correlation (R) between pairs of radiomics features was calculated to remove high-dimensional feature redundancy. A redundant feature was defined as the one with the smaller AUC of the ROC curve among two correlated radiomics features with R > 0.8. (3) The least absolute shrinkage and selection operator (LASSO) regression was used to filter combinations of radiomics features with high predictive efficacy. The λ value in the LASSO regression analysis was validated using 10-fold cross-validation (λ refers to the number of parameters included in the radiomics model). The optimal λ value is the one that results in the fewest and most stable model parameters. (4) The retained features and their corresponding weights were analyzed using multi-parameter logistic regression to derive the regression formula and calculate the Radiomics score (Rad_Score) for each patient. A stepwise backward multifactor logistic regression algorithm was used to construct the joint model by integrating imaging histology labels and clinical variables.

### Statistical analysis

SPSS v26.0 software (IBM, Armonk, NY, USA) was used for analyses. Measurement data conforming to normal distribution were expressed as mean ± SD, while data not conforming to normal distribution were expressed as M (Q1, Q3). Counting data were expressed as the number of cases or percentage. The comparison of measurement data conforming to normal distribution was performed using the two independent samples t-test, and for data not conforming to normal distribution, the Mann-Whitney U rank-sum test was used. Comparison of counting data was performed using the χ2 test. The Mann-Whitney U rank-sum test was applied to compare the differences in parameter values between the Ki-67 high expression and low expression groups. One-way logistic regression analysis was used to predict the independent factors influencing high Ki-67 expression. Based on the results of logistic regression, parameters with statistically significant differences were selected to build a comprehensive prediction model. ROC curves were plotted using MedCalc statistical software (Version 15.2.2), and the Delong test was used to compare the AUCs between different models. Since composite models based on clinical variables and radiomics labels have better predictive efficacy, this study constructed column line plots to visualize the individualized predictive models and tested model fit using calibration curves and the Hosmer-Lemeshow test (with the bootstrap method repeated 100 times). The predictive accuracy of the column-line diagrams was assessed using decision curve analysis (DCA), and correction curves were plotted using prediction probabilities and accuracy probabilities. A p-value < 0.05 was considered statistically significant.

## Results

### Clinical characteristics and PET-CT imaging of the enrolled cases

A total of 197 patients were enrolled, including 70 cases with low Ki-67 expression and 127 cases with high Ki-67 expression. The cohort consisted of 113 males and 84 females, with ages ranging from 5 to 85 years and a mean age of 52 years. Males predominated in the Ki-67 high-expression group (χ2 = 4.635, P = 0.031), and there was no significant difference in age between the two groups (t = 1.371, P = 0.172). Regarding systemic symptoms, the Ki-67 high-expression group was more likely to present with fever (χ2 = 4.246, P = 0.039). The Ki-67 high-expression group also showed a higher incidence of deep lymph node invasion and extra-nodal infiltration compared to the low-expression group; however, the difference was not statistically significant (both P > 0.05). There was no significant difference in smoking history between the two groups (all P > 0.05). In laboratory examinations, the differences in abnormal leukocytes, neutrophils, C-reactive protein, calcitoninogen, and blood sedimentation rates between the two groups were not statistically significant (all P > 0.05). The comparison results of clinical manifestations and laboratory examinations are presented in **Table 1**.

**Table 1:** Comparative results of clinical manifestations, laboratory tests and metabolic parameters in Ki-67 low expression group and Ki-67 high expression group.

	Ki-67 low expression (n=70)	Ki-67 high expression (n=127)	*P*
Gender (cases)			
male	33 (29.2%)	80 (70.8%)	0.031
Age (years)	54.2 ± 12.5	50.8 ± 18.6	0.172
Fever (cases)	13 (24.1%)	41 (75.9%)	0.039
Area of lymph node involvement (cases)			0.950
Superficial	19 (27.1%)	2 (72.9%)	
Deep	35 (27.6%)	96 (72.4%)	
Extra-lymph node involvement (cases)	20 (42.6%)	27 (57.4%)	0.249
smoking history(cases)	14 (30.4%)	32 (69.6%)	0.409
Abnormal WBC count (case)	17 (41.5%)	24 (58.5%)	0.373
Neutropenia (cases)	7 (41.2%)	10 (58.8%)	0.611
Elevated CRP	23 (29.9%)	54 (70.1%)	0.183
Elevated calcitonin	20 (27.4%)	53 (72.6%)	0.067
Elevated ESR	15 (44.1%)	19 (55.9%)	0.250
metabolic parameter			
T/MB of lymph nodes	2.9 (1.9,4.5)	7.3 (4.0,11.5)	<0.001
wbMTV_2.5_	32.1 (9.7,91.5)	52.4 (17.7,101.8)	0.064
wbTLG_2.5_	88.4 (26.1,198.73)	230.2 (66.4,655.0)	<0.001
wbMTV_42%_	30.1 (10.5,74.9)	36.1 (14.4,72.7)	0.382
wbTLG_42%_	33.8 (29.0,172.8)	197.0 (74.1,439.9)	<0.001

Continuous variables are expressed as median (P25, P75) and categorical variables are expressed as numbers (%).WBC, White Blood Cells; CRP, C Reactive Protein; ESR, Sedimentation.

### ^18^F-FDG uptake and its predictive efficacy

The T/MB of lymph nodes in the Ki-67 low expression group ranged from 0.9 to 11.7, while the T/MB in the Ki-67 high-expression group ranged from 1.2 to 32.9. T/MB, TLG2.5, and TLG42% in the Ki-67 high-expression group were significantly higher than in the Ki-67 low-expression group (all P < 0.05). The differences in MTV2.5 and MTV42% between the two groups were not statistically significant (see **Table 1**).

When T/MB had a cut-off value of 4.7, its area under the curve (AUC) for predicting high Ki-67 expression was 0.811 (95% CI: 0.759-0.863). When TLG2.5 had a cut-off value of 185.0, its predicted AUC for high Ki-67 expression was 0.670 (95% CI: 0.605-0.716). When TLG42% had a cut-off value of 127.9, its AUC for predicting high Ki-67 expression was 0.668 (95% CI: 0.592-0.753). The difference between the AUCs of TLG2.5 and TLG42% was not statistically significant (Z = 0.143, P = 0.886).

### Extraction and screening of radiomics parameters and creation of radiomics models and combined models

**Figure 3:**
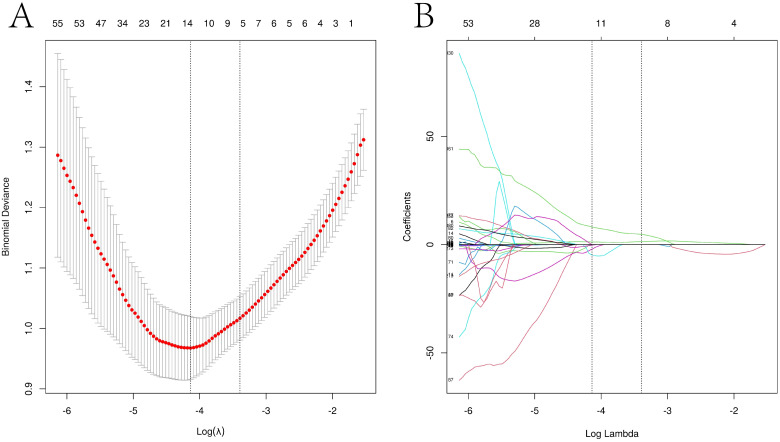
LASSO regression path diagram of each imaging histological feature and screening of the best imaging histological parameters Visualization of the Lasso model downscaling process. **(A)** Mean square error plot for 10-fold cross-validation. The vertical axis represents the mean square error value of each fold model, while the horizontal axis represents the logarithmic value of the parameter λ for each fold model. The Lasso model determines the optimal λ value based on the smallest mean square error, and the vertical dashed line in the graph indicates the optimal λ value. **(B)** Dimensionality reduction process of the Lasso model. The Lasso model constructs the penalty function using the optimal λ value and compresses the coefficients of unimportant variables to zero.

From the PET images, 1,225 radiomics features were extracted, of which 410 features with significant differences were selected. The optimal λ value of 0.016 was derived through LASSO regression, and based on this λ value, the dimensionality of the radiomics parameters was reduced to 13 (**Figure 3**).

These parameters were included in the logistic regression equation, and 5 parameters were found to be statistically significant. The formula for the Rad_Score was obtained:


$$\begin{array}{c} Rad\_Score=12.114-0.031\times original\_shape\_Maximum2DDiameterSlice\\ -0.001\times log.sigma.2.0.mm.3D\_firstorder\_Median\\ -17.757\times log.sigma.2.5.mm.3D\_glszm\_SizeZoneNonUniformityNormalized\\ +0.0003607\times original\_firstorder\_10Percentile\\ -0.001\times wavelet.HHL\_firstorder\_10Percentile \end{array}$$

(Formula notes: original original image, Laplace operator of log Gaussian filter with sigma as its specified parameter; low sigma emphasizes fine textures, high sigma values emphasize coarse textures. wavelet wavelet filtering; HHL denotes that the image is filtered with a high-pass function in the x-direction, high-pass function in the y-direction, and low-pass function in the z-direction.)

The Rad_Score of the Ki-67 high expression group was higher than that of the Ki-67 low expression group (both P < 0.001) (**Table 2**).

**Table 2:** Differences between Clinic-score, Rad_Score and Clinic_R in each group.

	Training cohort			Validation cohort		
	Ki-67 (0-49%)	Ki-67 (≥50%)	P	Ki-67 (0-49%)	Ki-67 (≥50%)	P
Clinic-score	-1.173 (-1.512,-0.495)	1.184 (-0.224,3.173)	<0.001	-0.760 (-1.671, -0.250)	1.426 (-0.647,2.940)	<0.001
Rad_Score	-0.172 (-1.127,0.446)	3.656 (1.879,5.345)	<0.001	-0.510(-1.114,0.395)	3.812(2.244,6.505)	<0.001
Clinic_R	-1.411 (-2.209, -0.547)	4.049 (1.323,6.832)	<0.001	-1.052 (-2.048, -0.714)	4.650 (1.319,8.301)	<0.001

Rad_Score: imaging histology score; Clinic: clinical parameter score; Clinic_R: combined score of Rad_Score and clinical parameters.

### Building clinical predictive models and combined

In the multivariate logistic regression analysis, fever and T/MB were identified as independent factors in predicting high Ki-67 expression (**Table 3**). Based on this study, the specific formula for the clinical model predicting high Ki-67 expression was:

**Table 3:** Multivariate analysis to predict high Ki-67 expression.

Characteristic	Multifactorial analysis	
	OR (95%CI)	P
Gender	2.72 (0.996, 7.947)	0.057
Fever	3.261 (1.075, 11.075)	0.044^∆^
T/MB	1.254 (1.148, 1.403)	0.000^∆^

^∆^P<0.05, Indicates a statistically significant difference in logistic regression analysis.


Clinic_Model=−2.552+1.182×Fever+0.226×T/MB

Each case obtained its Clinic-score by applying the above formula. In both the training and validation groups, the Clinic-score of the Ki-67 high-expression group was higher than that of the Ki-67 low-expression group (both P < 0.000) (see **Table 2** for details).

Building a combined model:


Combined_Model=−2.281+0.136×T/MB+2.084×Fever+1.035×R

The composite score for each case was calculated using the above formula. In both the training and validation sets, the composite score of the Ki-67 high-expression group was higher than that of the Ki-67 low-expression group (both P < 0.000). **Table 2** presents the composite scores for each case in the training and validation sets.

### Predictive performance of radiomics models, clinical models and combined models

In both the training and validation cohorts, the radiomics model demonstrated significantly better predictive ability for Ki-67 expression status compared to T/MB and the clinical model (Z = 3.002, P = 0.003; Z = 2.822, P = 0.005), with AUCs of 0.890 (95% CI: 0.825-0.955) and 0.883 (95% CI: 0.804-0.938), respectively. In addition, the AUC of the combined model in the training and validation cohorts was 0.921 (95% CI: 0.883-0.986) and 0.916 (95% CI: 0.862-0.980), respectively, which was not statistically different from the predictive ability of the radiomics model (Z = 1.324, P = 0.186). The sensitivity, specificity, and accuracy of the predictions for each model are presented in **Table 4**.

**Table 4:** Predictive power of the model.

	Training cohort				Validation cohort			
	AUC(95%CI)	Sensitivity(%)	Specificity(%)	Accuracy(%)	AUC(95%CI)	Sensitivity(%)	Specificity(%)	Accuracy(%)
Clinic model	0.827 (0.745-0.909)	0.721 (0.693-0.750)	0.854 (0.831-0.876)	0.767 (0.754-0.781)	0.803 (0.709-0.897)	0.681 (0.561-0.800)	0.835 (0.743-0.927)	0.732 (0.643-0.821)
Radiomics Model	0.890(0.825-0.955)	0.817 (0.806-0.827)	0.976(0.970-0.981)	0.872(0.865-0.879)	0.883 (0.804-0.938)	0.818 (0.724-0.912)	0.975 (0.926-0.997)	0.872 (0.810-0.934)
Combined model	0.921 (0.883-0.986)	0.869 (0.862-0.875)	0.951 (0.944-0.958)	0.897 (0.893-0.902)	0.916 (0.862-0.980)	0.870 (0.814-0.925)	0.950 (0.885-0.998)	0.898 (0.864-0.902)

### Creating column line diagrams

**Figure 4:**
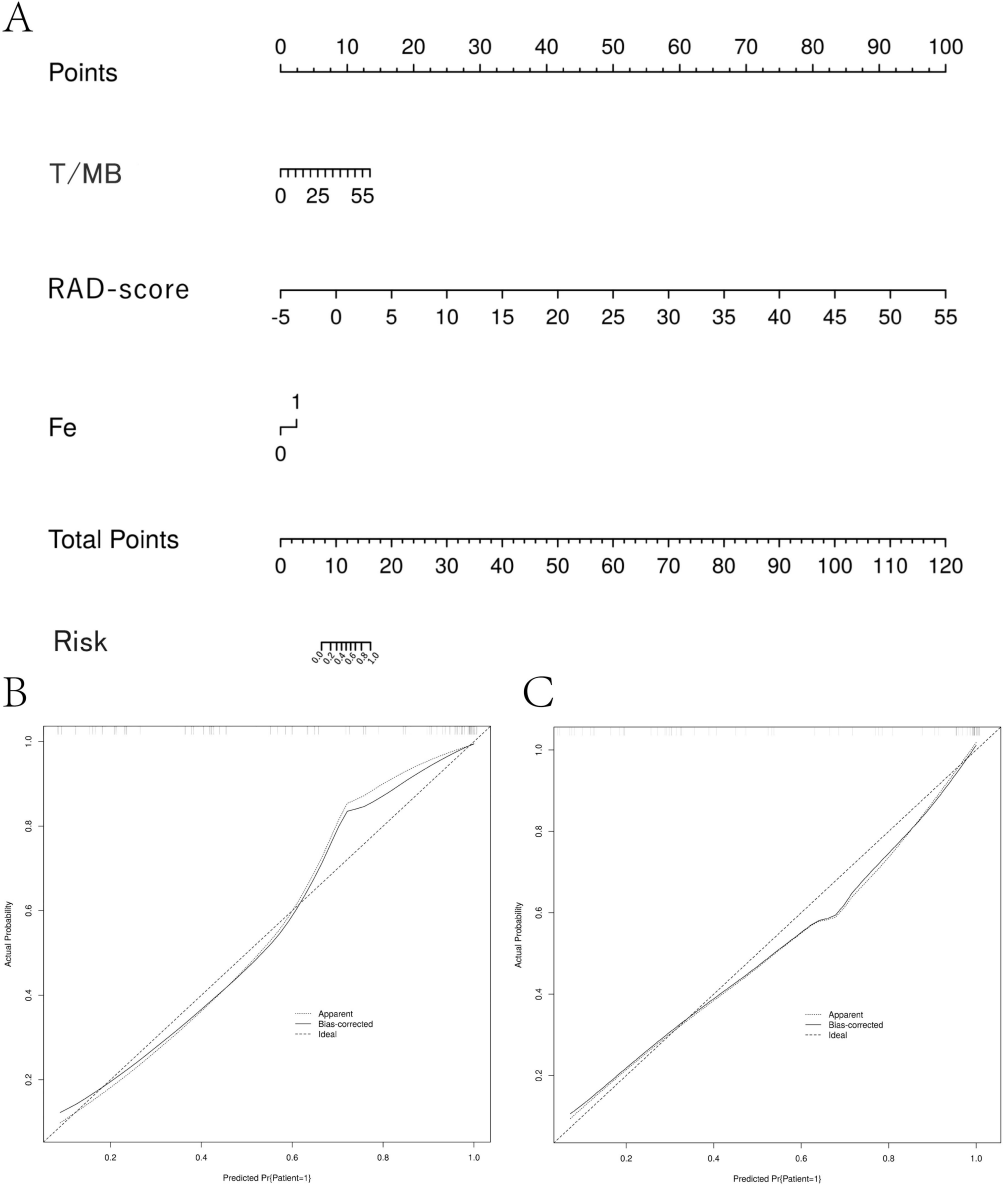
Column-line diagram and calibration curve creation. **(A)** The RAD-score value for each patient was calculated according to the imaging histology labeling formula. A vertical line was drawn for each variable to obtain the corresponding score, and then the scores for each factor were summed to get the total score. The risk probability of the patient having high Ki-67 expression was determined by drawing a vertical line according to the total score value. **(B, C)** Model calibration curves. The calibration curves show the predicted versus actual probability of high Ki-67 expression in non-Hodgkin’s lymphoma for the training cohort **(B)** and the validation cohort **(C)**.

Since the combined model had the highest absolute AUC, the risk value for each case diagnosed with high Ki-67 expression was calculated based on the weights of each factor (fever, T/MB, Rad_Score) in the combined model. The weight of each factor in predicting the disease was illustrated using column graphs. A column graph of the combined model was created using data from the training set (**Figure 4A**). The risk value for high Ki-67 expression for each case in the validation group was calculated based on the column-line graph created for the training group. The calibration curves for both the training and validation groups showed the correlation between the predicted and actual probabilities of cases within each group (**Figures 4B, C**). The Hosmer-Lemeshow test was applied to assess the goodness-of-fit (GOF) of the regression line to the test values. This revealed no statistical difference between the joint model in the training group (P = 0.942) and the validation group (P = 0.765), suggesting a good fit. The decision and calibration curves of the combined model demonstrated excellent fit and clinical utility.

## Discussion

The results of this study showed that the radiomics model not only demonstrated superior predictive ability in both the training and validation sets but also significantly outperformed the conventional metabolic parameter T/MB. Furthermore, the joint model, consisting of imaging radiomics features, fever, and T/MB, exhibited enhanced predictive ability.

In this study, the clinical data and ^18^F-FDG PET/CT images of 197 lymphoma patients were retrospectively analyzed. Ki-67 expression status was categorized with a 50% cut-off, where ≥50% was considered the high-expression group, characterized by high invasiveness, rapid proliferation, and a high degree of malignancy (40–43). To ensure a thorough and accurate diagnosis of systemic lesions in patients, the highest metabolic lesions in the body were used to measure SUVmax, TMTV, and TTLG. Additionally, following international recommendations, the mediastinal blood pool radioactivity was used as a reference background, and the ratio of SUV to mediastinal blood pool (T/MB) was applied as an evaluation index. This approach helped to minimize imbalances caused by variations in SUV resulting from differences in radiopharmaceutical injection amounts, waiting times for scanning, and individual blood circulation characteristics during the examination. This study also identified differences in gender, fever, T/MB, and TLG between lymphoma patients with different Ki-67 expression statuses. Specifically, the incidence of fever was higher in the high Ki-67 expression group than in the low Ki-67 expression group. This may be due to the vigorous metabolism of tumor cells with high proliferative activity, prompting the immune system to counteract tumor growth through mechanisms such as fever. Additionally, tumor cells with high proliferative activity are more likely to undergo necrosis, releasing inflammatory mediators such as tumor necrosis factor, which can induce fever. The results of a previous study have shown that the proliferative potential of lymphomas can be estimated using SUVmax from FDG-PET (43), but there are significant contradictions between different studies. Watanabe et al. found that all indolent lymphomas had a SUVmax <8, but 23% of aggressive lymphomas were also below this threshold, suggesting that 8 is insufficiently specific as a cut-off value (43). However, other studies used higher thresholds: Schoder’s team used SUVmax=10 as the critical value (21), while Luo Yao Guo et al. proposed an optimized threshold of SUVmax=12.14 (22). This heterogeneity in threshold selection has led to divergent conclusions between different studies on the correlation between FDG uptake and clinical aggressiveness, highlighting the limitations of a single parameter, SUVmax, in lymphoma staging. This study highlights the limited utility of PET-derived metabolic parameters (T/MB and TLG) in predicting Ki-67 expression in lymphoma, with T/MB demonstrating poor accuracy (<0.5) and TLG failing to serve as an independent predictive marker. These findings suggest that standalone PET metrics may inadequately reflect tumor proliferative activity, warranting further exploration of multimodal approaches for reliable Ki-67 assessment.

Although PET/CT provides a wealth of information, there is growing evidence that the naked eye often overlooks important details within medical images (17, 32), highlighting the need for a more objective and quantitative method to evaluate these images. Radiomics offers a method to objectively quantify tumor heterogeneity in medical images. This study emphasizes the superiority of ^18^F-FDG PET/CT over conventional CT/MRI for tumor delineation, particularly in addressing overestimation errors caused by irregular morphology or necrosis. By integrating metabolic activity (reflecting tumor biology) with structural imaging, PET enhances accuracy in defining tumor load and heterogeneity. The choice of PET for radiomics modeling is justified by its ability to capture biologically relevant texture features (e.g., proliferation, hypoxia), offering deeper insights into tumor heterogeneity compared to CT-derived radiomics alone (44). This study demonstrates the robust predictive potential of PET-based radiomics for evaluating Ki-67 expression and lymphoma aggressiveness. By integrating multi-dimensional features (morphology, density, texture) derived through advanced filtering and wavelet transformations, the radiomics model achieved high diagnostic accuracy (AUCs: 0.903–0.883), outperforming conventional PET metabolic parameters. Notably, the synergy of clinical data, metabolic parameters, and radiomics features further enhanced predictive performance, highlighting the importance of multimodal integration to account for biological and clinical interactions. These findings position radiomics as a promising tool for refining risk stratification in lymphoma management.

In recent years, with the rapid development of radiomics technology, the application of radiomics in predicting the Ki-67 expression level of tumors has garnered increasing attention. In a lung cancer study, a model was established to predict the Ki-67 expression level by extracting and analyzing texture features from CT images. This model demonstrated high predictive accuracy and provided strong support for the clinical diagnosis and treatment of lung cancer (34). Additionally, ^18^F-FDG PET/CT-based radiomics has gradually gained traction in predicting molecular subtypes and Ki-67 expression levels in breast cancer. A study involving 134 breast cancer patients showed that a composite model combining PET/CT radiomics and clinical factors could more accurately predict molecular subtypes and Ki-67 expression levels, providing a solid foundation for individualized treatment (45). Beyond lung and breast cancers, PET/CT radiomics has shown promise in predicting Ki-67 expression levels in other tumors, such as renal and cranial tumors (35, 46). PET radiomics also holds potential for predicting Ki-67 expression in non-Hodgkin’s lymphoma. However, there are still limitations in current studies within this field, including differences in sample sizes, research methods, and image processing techniques, which contribute to variability in results. While some studies have achieved positive preliminary validation, the lack of large-scale clinical validation means their feasibility and effectiveness in practical applications require further evaluation. Moreover, combining radiomics information with patient-specific conditions to develop personalized treatment plans remains an area that needs further exploration and refinement.

There are several major limitations in this study: (1) this is a retrospective, single-center study, which may introduce bias in patient selection and needs to be validated by multicenter studies; (2) we did not follow up with the patients, and this limitation should be addressed in future research; (3) only one imaging modality was used. The inclusion of additional modalities, such as the ADC value from MRI examinations of lymphoma, could provide valuable insights. In future studies, we aim to combine macroscopic and microscopic parameters from multimodal imaging to offer comprehensive information for lymphoma diagnosis and treatment from different perspectives; (4) due to the variability of PET measurements, interference of technical variability on model performance needs to be reduced in the future through rigorous standardized protocols (e.g., device calibration, feature stability testing), dynamic metabolic assessment, and multi-center large sample validation.

## Conclusions

In this study, the radiomics model based on ^18^F-FDG PET images demonstrated higher predictive value for Ki-67 high expression status in non-Hodgkin’s lymphoma. The combined model, which integrates both macro- and micro-parameters from imaging and clinical features, provides information from multiple perspectives, enabling more accurate prediction of Ki-67 high expression. These models can aid in triaging patients, especially in light of increasing workloads, and offer supportive opinions to assess lymphoma aggressiveness, particularly in cases where IHC examination may be inconclusive due to poor pathology sampling or other factors.

To further understand the role of radiomics in distinguishing tumor heterogeneity, prospective studies are needed to explore the biological and clinical differences in lymphomas of various histopathological subtypes and their corresponding therapeutic approaches. It is hoped that, in the near future, more studies will confirm the potential role of ^18^F-FDG PET radiomics in selecting stable and reliable imaging biomarkers. Future research should also focus on the use of different imaging agents.

## Data Availability

The original contributions presented in the study are included in the article/supplementary material. Further inquiries can be directed to the corresponding authors.
